# Bifidobacterium Against COVID-19: A Mother and Her Newborn’s Gut Microbiome

**DOI:** 10.7759/cureus.60038

**Published:** 2024-05-10

**Authors:** Sabine Hazan, Megan Smith, Skye Lander, Abby Carlson, Camila Walters

**Affiliations:** 1 Research, ProgenaBiome, Ventura, USA; 2 Biomedical Sciences, California University of Science and Medicine, Colton, USA; 3 Research and Development, ProgenaBiome, Ventura, USA; 4 Anesthesiology, Vanderbilt University Medical Center, Nashville, USA

**Keywords:** vitamin c, newborn, bifidobacterium, breastfeed, gut microbiome

## Abstract

Several treatments and preventive measures for SARS-CoV-2 were studied during the pandemic, but few focused on the neonatal gut microbiome and its role in the setting of COVID-19. This case report is unique because it describes the gut microbiomes of a mother and her newborn, who both contracted COVID-19 shortly after the baby’s birth. In this prospective study, on day 11 postpartum, both the newborn and mother (38 years old), of white race/ethnicity, were exposed to a COVID-19-positive person. After exposure, the mother received a 40,000 IU bolus of vitamin D orally and started a five-day course of high-dose vitamin C (10,000 mg daily), after which she continued her daily combination of vitamins C, D, and zinc pill with probiotic skyr yogurt and manuka honey. Stool specimens and DNA were extracted, quantitated, and normalized from the mother and the newborn for downstream library fabrication utilizing shotgun methodology. Baseline Bifidobacteria level for the mother was 1.5% which increased to 19% on day 15 postpartum after testing positive for COVID-19 and taking vitamin C. Neonatal Bifidobacteriasteadily increased regardless of COVID-19 infection. We propose that the disease course was altered by maternal supplementation of vitamins C and D and zinc, which may have increased *Bifidobacterium *levels and led to improved outcomes for both patients.

## Introduction

As of June 2023, the COVID-19 pandemic has seen 767 million confirmed cases and almost 7 million deaths worldwide [1]. A signature microbiome was reported in those who survived which focused on the presence of Bifidobacteria [2]. Several species of the genus *Bifidobacterium* of the phylum Actinobacteria have been shown to improve immune function in vivo [3], and supplementation of specific vitamins such as C and D have been shown to increase gut Bifidobacteria levels [4,5]. Formation of the early neonatal gut microbiome depends on several factors, including vaginal delivery versus cesarean section, feeding by breast milk versus formula, age of weaning, malnutrition, as well as congenital conditions and environmental factors [6]. Here, we present a case report of a mother and her newborn infected with COVID-19 within two weeks of the baby’s birth. This report demonstrates the impact of Bifidobacteria on the COVID-19 disease course and the growth of Bifidobacteria over six months of breastfeeding in the setting of maternal supplementation of vitamins C and D and zinc.

Infection with SARS-CoV-2 has been shown to affect the gut microbiome through several mechanisms leading to dysbiosis manifesting as diarrhea, vomiting, abdominal pain, anorexia, and more, even in mild cases of COVID-19. Some mechanisms proposed to be implicated in the disease course are the translocation of gut bacteria to systemic circulation and loss of commensal immunomodulatory organisms including Bifidobacteria leading to long-term dysbiosis in COVID-19 patients [7,8]. Several mice and human studies have examined the implications of Bifidobacteria with the gut-lung axis in the setting of respiratory disease including COVID-19, proposing protective effects from increased levels of several members of the *Bifidobacterium* genus [2,9,10].

Early life gut microbiome development has long-term health implications, including immune function and development [11]. Bifidobacteria colonization in the breastfed infant gut is centered around human milk oligosaccharides (HMOs). HMOs are carbohydrates produced in maternal breast milk that are not broken down by enzymes in the enteric brush border but fermented by Bifidobacteria and other anaerobes [12]. Bifidobacteria colonization and their beneficial effects are strongly linked to vertical transmission through breast milk. This may help explain why breastfed infants have better health outcomes when exposed to pathogens than bottle-fed infants [13].

## Case presentation

Pregnancy and maternal health

The patient, a 38-year-old white woman, provided written informed consent for herself and her newborn (Salus IRB-approved protocol #20110) at Progenabiome. Pregnancy was complicated by diet-controlled gestational diabetes without fetal growth restriction, macrosomia, or other abnormalities. Prenatal ultrasounds were within normal limits. The mother’s past medical history included papillary thyroid carcinoma treated by thyroidectomy and I-131 therapy in remission. The mother was on levothyroxine as a hormone replacement at doses designed to keep further carcinoma growth down (thyroid-stimulating hormone <0.015 mIU/L). A basic metabolic panel, complete blood count, coagulation studies, and liver function tests were all within normal limits. The mother was unvaccinated against SARS-CoV-2. The mother was regularly taking a daily combination pill with 3,000 mg of vitamin C, 3,000 IU of vitamin D, and 50 mg of zinc.

Birth and neonatal care

The mother was at G3P3 at the time of delivery. As there was concern with advanced maternal age and decreased fetal movement, vaginal delivery was induced at 38 weeks and one day of gestation via intravenous (IV) oxytocin and a Foley bulb, resulting in a female newborn. Placenta appearance and pathology report were within normal limits. The newborn, of white race/ethnicity, developed neonatal respiratory distress syndrome due to prolonged induction time (16 hours and 40 minutes), increased IV fluids, lack of pushing, and lack of thoracic squeezing of the newborn. The newborn was treated with one dose of surfactant and high-flow oxygen via nasal cannula for two days. On day three, the newborn was breathing well on room air but stayed in the hospital for another two days to receive light therapy for neonatal jaundice. No congenital abnormalities were noted and the child had not developed chronic medical conditions in the first two years of life.

COVID-19 and supplementation

During labor induction, the SARS-CoV-2 polymerase chain reaction (PCR) nasal swab was negative and the mother was asymptomatic for respiratory infection. In this prospective study, at day 11 postpartum, both the newborn and mother (38 years old), of white race/ethnicity, were exposed to a COVID-19-positive person. After exposure, the mother received a 40,000 IU bolus of vitamin D orally and started a five-day course of high-dose vitamin C (10,000 mg daily), after which she continued her daily combination of a vitamins C, D, and zinc pill with probiotic skyr yogurt and manuka honey. On day 14 postpartum, the mother began to experience upper respiratory infection symptoms, including congestion, sore throat, myalgia, abnormal skin sensations, shortness of breath, severe fatigue, coughing, and mild chest pain. The mother had a positive PCR test for SARS-CoV-2 on day 15 postpartum. A respiratory panel was otherwise negative for both the mother and the newborn. The mother was treated with ivermectin 12 mg on days 19 and 26 postpartum. Symptoms were vastly improved within seven days of COVID-19 infection (day 22 postpartum), with no symptoms on day 10 of COVID-19 infection (day 25 postpartum).

The newborn sneezed five times on day 15 postpartum with a positive PCR test for SARS-CoV-2 but was asymptomatic the next day. The newborn was exclusively fed breast milk via minimal direct breastfeeding and expressed milk. The newborn also received vitamin D 400 IU daily and simethicone 0.3 mL as needed. The mother started breastfeeding from day one postpartum and did not wash her newborn for 48 hours, both of which have been shown to improve immune system function in newborns [14,15].

Stool collection and microbiome sequencing

The newborn’s stools were collected on postpartum days 1, 4, 14, 22, and month 6. The mother’s stools were collected on days 2, 5, 14, 22, and 30. Next-generation sequencing was performed on both stool samples. From stool specimens, DNA was extracted, quantitated, and normalized for downstream library fabrication utilizing shotgun methodology. Prepared and indexed libraries were subsequently pooled and sequenced on the Illumina NextSeq 550 System. Sample FASTQ files were analyzed with downstream bioinformatics to profile the microbial communities from metagenomic sequencing data. Diversity analyses were performed to characterize the intestinal microbiota of study participants, and the Simpson and Shannon Diversity Indexes were used to calculate gut microbiome diversity and abundance.

**Figure 1:**
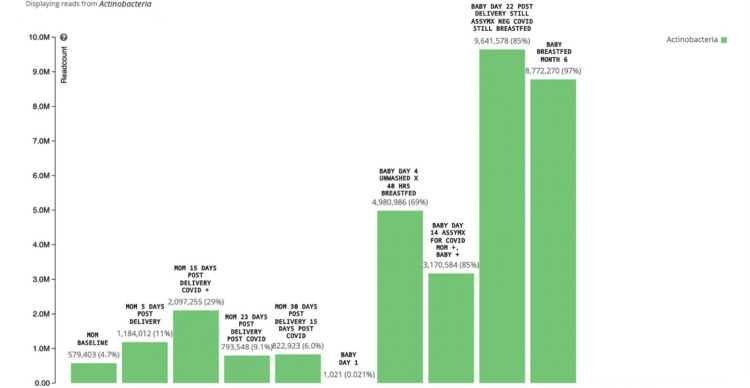
Actinobacteria relative abundance in the mother and newborn. Baseline Actinobacteria level for the mother was 4.7% which increased to 29% on day 15 postpartum after testing positive for COVID-19 and taking vitamin C. Neonatal Actinobacteria steadily increased regardless of COVID-19 infection.

**Figure 2:**
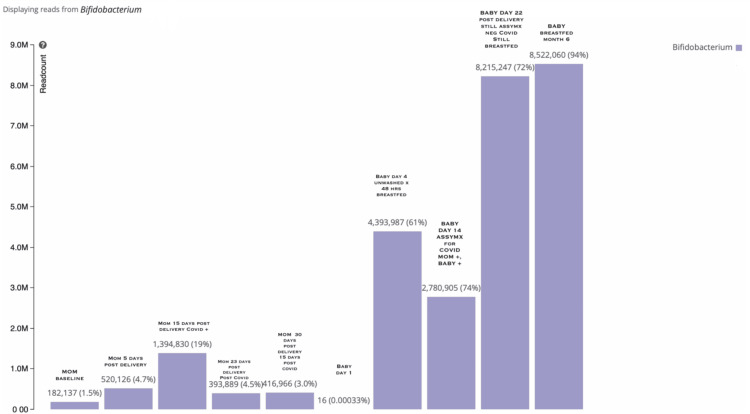
Bifidobacteria relative abundance in the mother and newborn. Baseline Bifidobacteria level for the mother was 1.5% which increased to 19% on day 15 postpartum after testing positive for COVID-19 and taking vitamin C. Neonatal Bifidobacteria steadily increased regardless of COVID-19 infection.

At the phylum level, gut Actinobacteria increased in the mother and the newborn. In the mother, Actinobacteria increased to 29% on day 15 postpartum after testing positive for COVID-19 and taking vitamin C (Figure 1). In the breastfed newborn, Actinobacteria increased to 87% at six months of age. Concomitantly, the relative abundance of Bifidobacteria level for the mother increased to 19% from 1.5% on day 15 postpartum. Neonatal Bifidobacteria steadily increased regardless of COVID-19 infection (Figure 2).

## Discussion

The mother had elevated Bifidobacteria on day 15 postpartum, likely from high doses of vitamin C that she started on day 11 [5]. Bifidobacteria levels in the mother dropped during COVID-19 most likely from the virus creating extra bifidophage. Her Bifidobacteria level stabilized to baseline at one month. Although there is limited data on the maternal postpartum gut microbiota, studies are starting to emerge; for example, a review of 27 studies found that Firmicutes was the predominant phylum in the early (<6 weeks) and late postpartum (6 weeks to 1 year), while in early postpartum, Bacteroides was the predominant genus [16]. To our knowledge, this is the first study to focus on gut *Bifidobacterium* in maternal postpartum.

Neonatal bifidobacteria was low at baseline with a relative abundance of 0.033E-2%, which increased to 61% on day four postpartum with breastfeeding and without being washed. It then increased to 74% on day 14, 72% on day 22, and 94% by month six. The newborn was diagnosed with neonatal respiratory distress syndrome at birth, lasting one day, but recovered before COVID-19 infection and had only minor symptoms as of COVID-19 infection. This is likely due to the mother’s supplementation and breastfeeding increasing the newborn’s Bifidobacteria.

Vitamins C and D have been shown to pass into breast milk and have positive impacts on neonatal immunity in addition to the passive immunity breast milk normally provides [17]. This is the first case to show the positive effects of vitamin C, D, and zinc supplementation not only per se but also on the gut microbiomes of a mother and her newborn with COVID-19 infection, resulting in favorable health outcomes. This is also the first case to demonstrate the effect of breastfeeding on the infant microbiome in the setting of COVID-19. In addition to other health benefits, breastfeeding in combination with vitamins may increase gut Bifidobacteria levels, providing immune benefits to newborns and other infants. Further studies should look at Bifidobacteria levels pre- and post-delivery, while babies are breastfed versus formula-fed, and with and without maternal vitamin supplementation. There are considerations to be made regarding cesarean section versus vaginal delivery impacts on the gut microbiome as well. Further studies should also assess normal levels of Bifidobacteria in newborns, and whether or not relative levels of Bifidobacteria have positive or negative impacts on infant health and developmental outcomes.

## Conclusions

This is the first case to show the effects of vitamin C, D, and zinc supplementation on the gut microbiomes of a mother and her newborn with COVID-19 infection, resulting in favorable health outcomes. This study suggests that the disease course may have been altered by maternal supplementation of vitamins C and D and zinc, which may have increased *Bifidobacterium* levels and led to improved outcomes for both patients. Future studies are needed to confirm these findings.
